# Trifluoromethylated hydrazones and acylhydrazones as potent nitrogen-containing fluorinated building blocks

**DOI:** 10.3762/bjoc.19.127

**Published:** 2023-11-15

**Authors:** Zhang Dongxu

**Affiliations:** 1 Department of Fire Protection Engineering, China Fire and Rescue Institute, Beijing 102202, P. R. of China

**Keywords:** acylhydrazones, difluoromethylation, dihydropyridazine, fluorinated building blocks, hydrazones, imidazolidines, pyrazoles, pyrazolidines, pyrazolines, trifluoromethylation

## Abstract

Nitrogen-containing organofluorine derivatives, which are prepared using fluorinated building blocks, are among the most important active fragments in various pharmaceutical and agrochemical products. This review focuses on the reactivity, synthesis, and applications of fluoromethylated hydrazones and acylhydrazones. It summarizes recent methodologies that have been used for the synthesis of various nitrogen-containing organofluorine compounds.

## Introduction

The introduction of fluorine into pharmaceuticals, agrochemicals, and materials can significantly enhance lipophilicity and metabolic stability compared to nonfluorinated compounds [1–5]. At present, about 300 drug molecules and over 400 pesticides on the market contain at least one fluorine atom [6–7]. Therefore, the development of novel and effective synthetic methodologies for the synthesis of organofluorine compounds has become a major research focus.

The use of difluoromethylating and trifluoromethylating reagents is a popular approach applied to prepare di/trifluoromethyl-containing molecules [8–18]. Also the reaction of diverse di/trifluoromethyl-containing building blocks offers another mainstream approach to introducing fluorine. Among these, di/trifluorodiazoethane [19–22], trifluoromethyl aldimines [23–25], trifluoroacetimidoyl halides [26], and fluoroalkyl *N*-sulfonyl hydrazones [27] have emerged as powerful nitrogen-containing fluorinated building blocks that have been used to construct organofluorine derivatives. To the best of our knowledge, the synthetic applications of fluoromethylated hydrazones and acylhydrazones as useful building blocks, has not yet been summarized. Hence, the present review highlights recent advancements enabling the synthesis of diverse di/trifluoromethyl-containing molecules by using di/trifluoromethylated hydrazones, acylhydrazones, and their related compounds.

## Review

### Trifluoroacetaldehyde hydrazones

Hydrazones possess diverse biological and pharmacological properties and have been employed in the treatment of several diseases [28–30]. They have also been applied in the field of materials science, especially for the synthesis of metal and covalent organic frameworks, dyes, hole-transporting materials and sensors, and in dynamic combinatorial chemistry [31], indicating a wide applicability. Hydrazones can be regarded as electrophilic and nucleophilic imine equivalents, and thus they represent valuable and versatile building blocks in synthetic chemistry [32–36].

**Scheme 1:**
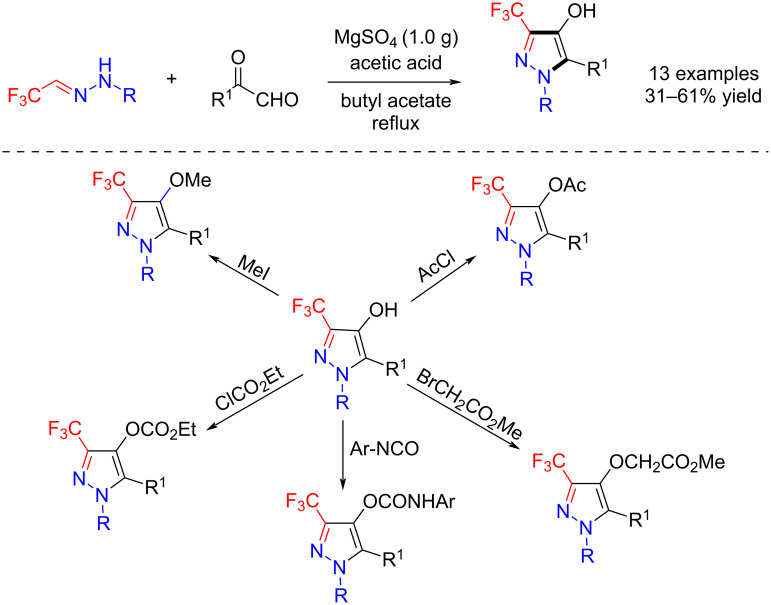
Synthesis of trifluoromethylpyrazoles from trifluoroacetaldehyde hydrazones.

Trifluoroacetaldehyde hydrazones can be regarded as an equivalent of fluorine-containing azomethine imines in the presence of Brønsted acid. In their pioneering research, Tanaka et al. reported the [3 + 2] cycloaddition reactions of trifluoroacetaldehyde hydrazones and glyoxals to give 4-hydroxy-3-trifluoromethylpyrazoles. The resultant pyrazoles containing a free 4-hydroxy group were easily converted to a variety of other derivatives in good yields [37] (Scheme 1).

**Scheme 2:**
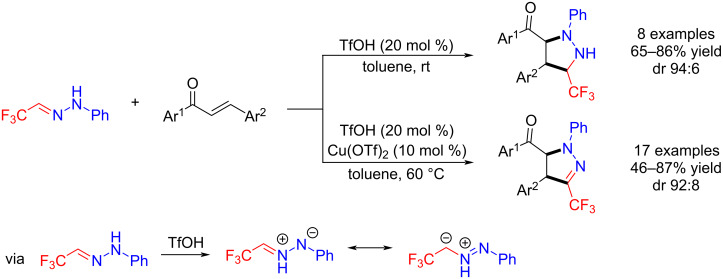
Synthesis of polysubstituted pyrazolidines and pyrazolines.

Later, Wu et al. described a diastereoselective 1,3-dipolar cycloaddition of trifluoroacetaldehyde hydrazones with α,β-ethenyl ketones to obtain polysubstituted pyrazolidines and pyrazolines. These reactions were carried out under two different sets of conditions [38] (Scheme 2).

**Scheme 3:**
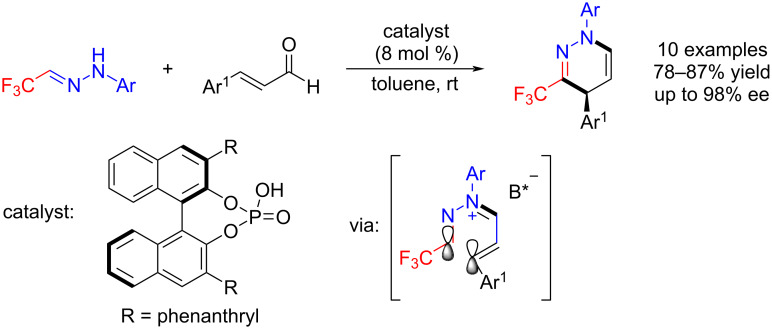
Asymmetric synthesis of 3-trifluoromethyl-1,4-dihydropyridazines reported by Rueping et al. [39].

Moreover, a chiral Brønsted acid-catalyzed asymmetric 6π electrocyclization of trifluoroacetaldehyde hydrazones for the synthesis of enantiomerically enriched 3-trifluoromethyl-1,4-dihydropyridazines was first developed by Rueping et al. [39]. The strategy involves chiral ion pairs and provides a good basis and scope for further extensions and explorations [39] (Scheme 3).

**Scheme 4:**
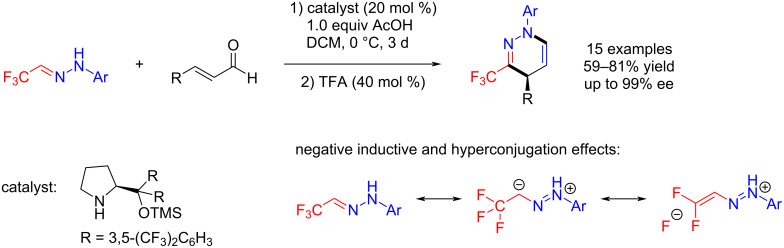
Synthesis of 3-trifluoromethyl-1,4-dihydropyridazine with Brønsted acid-assisted Lewis base catalysis.

Based on the work by Wu et al. and extending their previous work, Rueping and co-workers explored the effects of fluorine in organocatalytic reactions. They developed an asymmetric Brønsted acid–Lewis base catalysis, for the synthesis of trifluoromethylated dihydropyridazines under simple reaction conditions and the chemistry displayed very good enantioselectivities and high functional group tolerance (Scheme 4) [40].

**Scheme 5:**
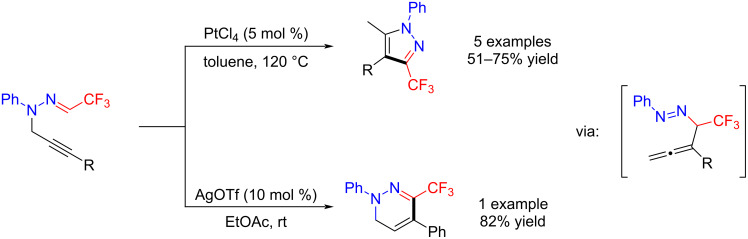
Synthesis of CF_3_-pyrazoles and CF_3_-1,6-dihydropyridazines.

Zhan et al. reported an efficient and highly selective method for the synthesis of CF_3_-pyrazoles and CF_3_-1,6-dihydropyridazines from readily available trifluoromethylated *N*-propargylic hydrazones. These reactions proceed through intermediate diazoallenes, and were conducted with catalytic PtCl_4_ [41] or AgOTf [42] (Scheme 5).

Their study explored the effects of fluorine through reactions with trifluoroacetaldehyde hydrazones. Jasiński et al. demonstrated that the CF_3_ group offered an appropriate electronic balance through experimental spectral analysis and computational DFT methods, and the hydrazones could be readily used to provide convenient access to azo tautomers under the acidic conditions [43].

**Scheme 6:**
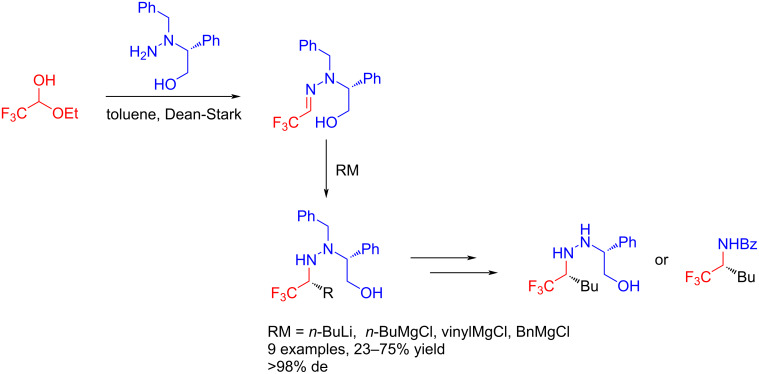
Asymmetric reactions of trifluoromethylimines with organometallic reagents.

The C=N motif within hydrazones gives them both electrophilic and nucleophilic character. In 2005, Brigaud et al. developed a highly stereoselective method for the synthesis of α-trifluoromethylamines with organometallic reagents to extend the asymmetric methodologies of trifluoroacetaldehyde hydrazones [44] (Scheme 6).

**Scheme 7:**
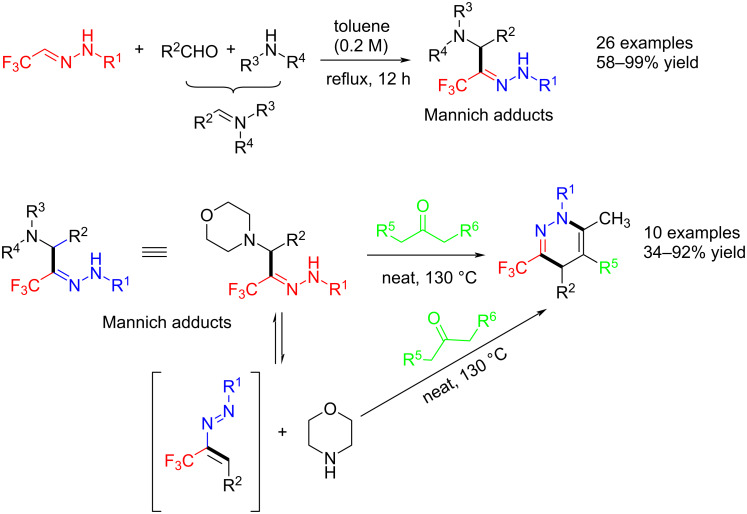
Mannich-type reaction of trifluoroacetaldehyde hydrazones.

El Kaim and Jia reported a Mannich-type reaction of trifluoroacetaldehyde hydrazones with formaldehyde and aromatic aldehydes to obtain valuable starting materials for the generation of other trifluoromethyl-substituted heterocycles. The study demonstrated that the electron-withdrawing property of the trifluoromethyl group is key to this coupling reaction [45] (Scheme 7).

### Trifluoromethylated hydrazonoyl halides

Hydrazonoyl halides, which offer a reactive 1,3-dipole, can easily be transformed to nitrile imines in the presence of a base, and they have shown to be useful building blocks for the synthesis of heterocycles [46–47]. The resultant heterocycles bearing a fluorine or fluorine-containing group have been used in a broad array of pharmaceutical applications [48–49]. The use of di/trifluoromethylated hydrazonoyl halides as building blocks for the synthesis of di/trifluoromethylated organic molecules is equally attractive and proven to be important.

**Scheme 8:**
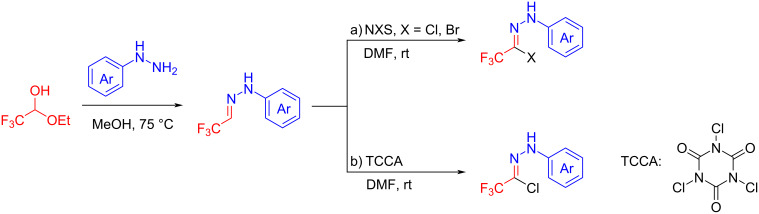
Synthesis of trifluoromethylated hydrazonoyl halides.

Generally, the reaction of trifluoroacetaldehyde hydrazones with *N*-chloro- and *N*-bromosuccinimide is used to prepare trifluoromethylated hydrazonoyl halides (Scheme 8a), or alternatively trichloroisocyanuric acid (TCCA) can be used as a chloride source for the preparation of these compounds [50] (Scheme 8b).

**Scheme 9:**
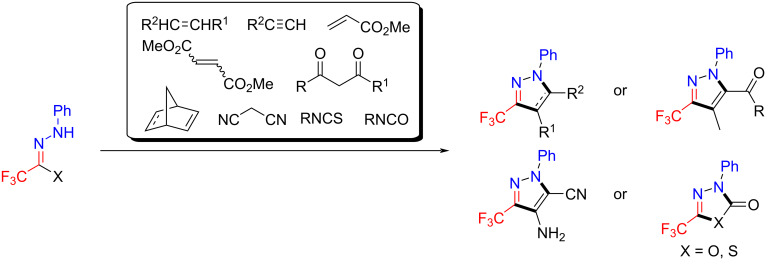
Early work of trifluoromethylated hydrazonoyl halides.

As early as in 1982, the reactivity of trifluoromethylated hydrazonoyl halides in the presence of base has been systematically studied by Tanaka et al. The trifluoromethylated hydrazonoyl halides, as the precursors of trifluoroacetonitrile imine, are highly versatile in that they react with olefins, acetylenes, dimethyl fumarate, dimethyl maleate, β-diketones, β-keto esters, bicyclic olefins, and potassium isothiocyanate and isocyanate affording the corresponding trifluoromethyl-containing compounds, generally with good yields [51–58] (Scheme 9).

**Scheme 10:**
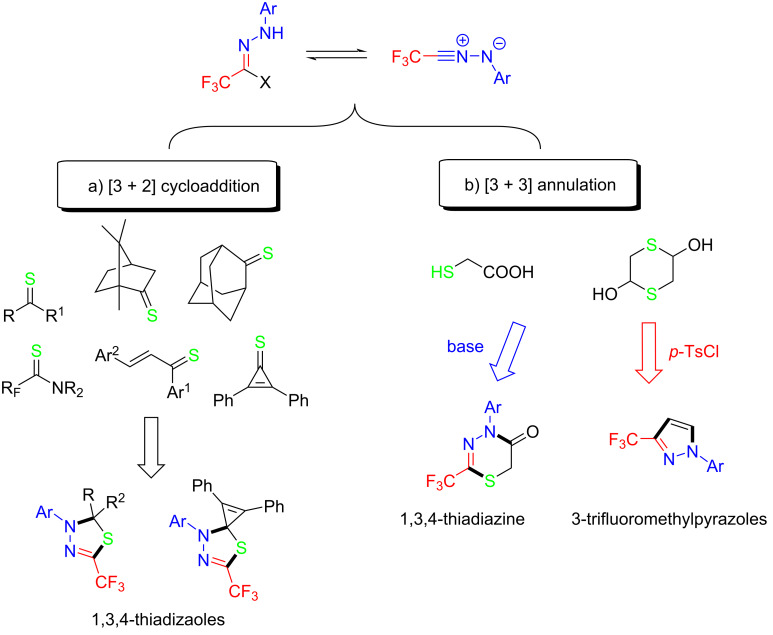
[3 + 2]/[3 + 3] Cycloadditions of trifluoromethylated hydrazonoyl halides.

Thioketones, thiochalcones, and tertiary thioamides react as C=S super dipolarophilic agents. Jasiński et al. reported that these thiocarbonyl compounds react with trifluoromethylated hydrazonoyl halides to give trifluoromethylated 1,3,4-thiadiazoles via regioselective [3 + 2] cycloadditions [59–63] (Scheme 10a). Similarly, trifluoroacetonitrile imine reacted with mercaptoacetaldehyde and mercaptocarboxylic acids to generate fluorinated 1,3,4-thiadiazines with good yields via a [3 + 3] annulation [64] (Scheme 10b).

Meanwhile, mercaptoacetaldehyde as a surrogate of acetylene reacted with trifluoroacetonitrile imine to form 1-aryl-3-trifluoromethylpyrazoles, followed by a series of cascade annulation/dehydration/ring contraction reactions when treated with *p*-TsCl [65] (Scheme 10b).

**Scheme 11:**
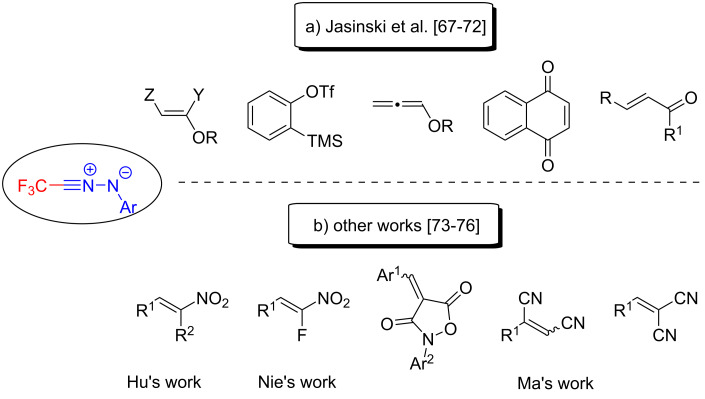
Substrate scope for [3 + 2] cycloadditions with trifluoroacetonitrile imines reported by Jasiński’s team and other groups.

The chemistry of pyrazoles with a fluorine or a fluoroalkylated group has attracted a significant level of attention, and many trifluoromethylated pyrazoles have been used in medicinal products or in pesticides [66]. The [3 + 2] cycloaddition reactions are considered among the most powerful tools for the synthesis of versatile fluoroalkylated pyrazoles. Enol ethers, 1,4-naphthoquinones, *o*-trimethylsilylphenyl triflate and chalcones have all been reacted with fluorinated nitrile imines to give a series of fluoroalkylated pyrazoles by Jasiński’s team [67–72] (Scheme 11a).

Subsequently, Hu et al., Nie et al., and Ma et al. have all independently reported practical methods, which extended the structural scope of such dipoles. This has allowed the synthesis of trifluoromethylpyrazoles by a range of regioselective [3 + 2] cycloadditions of trifluoroacetonitrile imines with electron-poor olefins [73–76] (Scheme 11b).

**Scheme 12:**
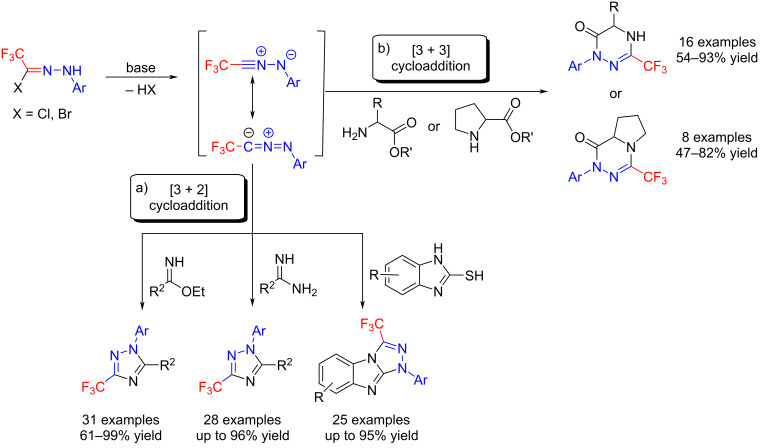
Synthesis of trifluoromethylated 1,2,4-triazole and 1,2,4-triazine derivatives.

Moreover, the trifluoromethylated 1,2,4-triazoles were synthesized with excellent regioselectivities in [3 + 2] cycloaddition reactions of trifluoromethylated hydrazonoyl chlorides with imidates, amidine and 1*H*-benzo[*d*]imidazole-2-thiols, all of which were individually reported by Wang, Deng and Cai, respectively [77–79] (Scheme 12a). Meanwhile, the Jasiński group turned their attention to the [3 + 3] cycloaddition of α-amino esters and trifluoromethylated hydrazonoyl halides and demonstrated the efficient synthesis of trifluoromethylated 1,2,4-triazine derivatives [80] (Scheme 12b).

**Scheme 13:**
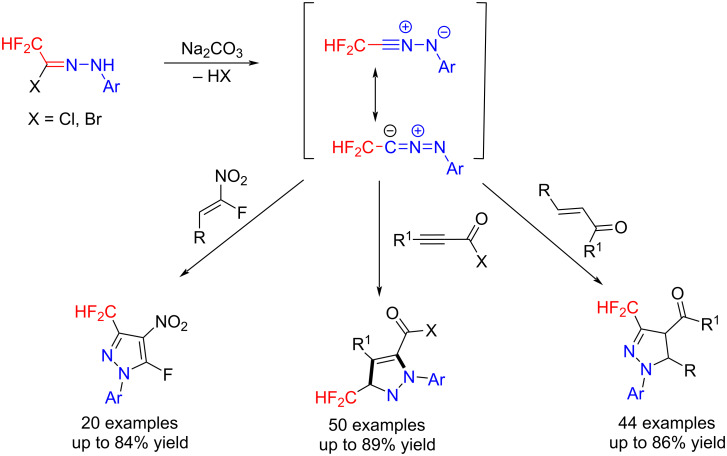
[3 + 2] Cycloadditions of difluoromethylated hydrazonoyl halides.

Difluoromethylated compounds play an indispensable role in drug discovery and design since the hydrogen atom can act as lipophilic hydrogen-bond donor and act as a bioisostere for the alcohol moiety [81–83]. Thus, many effective difluoromethylation strategies have been developed in recent years. Difluoroacetohydrazonoyl bromides were chosen as fluorinated building blocks for the synthesis of difluoromethylated pyrazole derivatives by such [3 + 2] cycloaddition reactions [73,84–85] (Scheme 13).

These studies therefore emphasize that fluoromethylated nitrile imines are versatile building blocks for [3 + 2] and [3 + 3] cycloaddition reactions and indicate their potential offering an efficient approach to fluoroalkylated heterocycles in drug design.

### Trifluoromethylated acylhydrazonoes

Acylhydrazones are a well-established class of organic compounds with the –CONH–N=CH– structure, and some variants show potential pesticidal and pharmacological activities [86–87]. Acylhydrazones can exist in either *E* or *Z* forms in solution, and they can exhibit good optical properties for applications as photoswitches, in luminescence sensing, and as metallo-assemblies [88–89]. In organic synthesis, acylhydrazones have served as stable imine equivalents to react with various nucleophilic reagents [90].

**Scheme 14:**
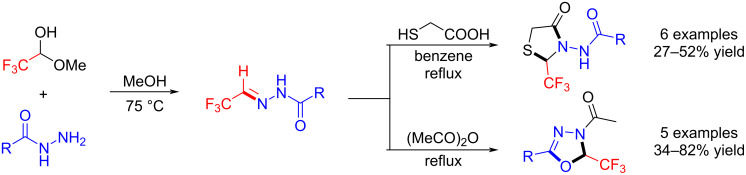
Preparation and early applications of trifluoromethylated acylhydrazones.

In 2014, Heimgartner et al. first developed the condensation reaction of a commercially available fluoral hemiacetal with acylhydrazides to yield trifluoromethylated acylhydrazones, and these fluorinated compounds underwent heterocyclization reactions with mercaptoacetic acid and acetic anhydride leading to trifluoromethylated 1,3-thiazolidin-4-ones and 3-acetyl-2,3-dihydro-1,3,4-oxadiazoles, respectively. It was found that the C=N reactivity of the trifluoromethylated acylhydrazones is similar to that of other nitrogen-containing fluorinated building blocks [91] (Scheme 14).

**Scheme 15:**
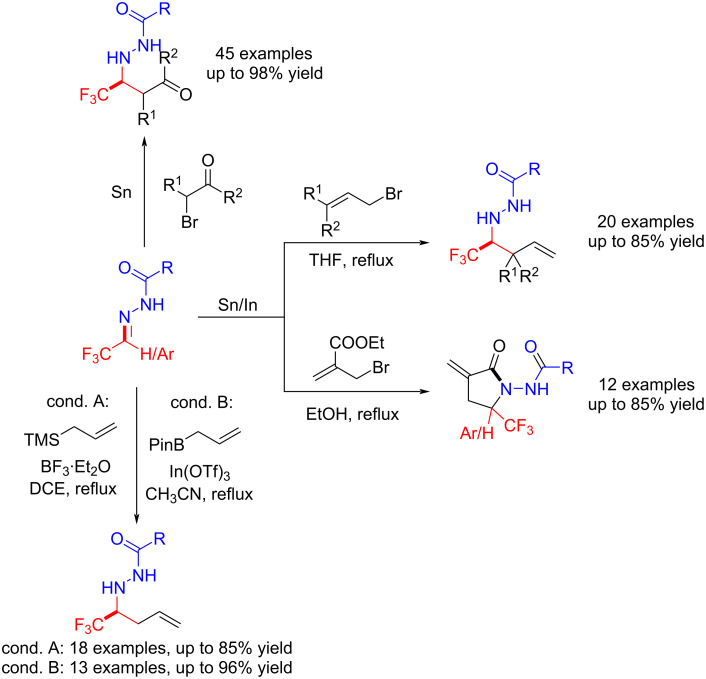
1,2-Nucleophilic addition reactions of trifluoromethylated acylhydrazones.

Inspired by previous accounts and this work [92–93], Hu et al. explored 1,2- nucleophilic addition reactions of trifluoromethylated acylhydrazones with organometallic reagents for the synthesis of trifluorinated homoallylic acylhydrazines [94–98], trifluorinated α-methylene-γ-lactams [99–100], and β-trifluoromethyl-β-acylhydrazonyl carbonyl compounds [101] (Scheme 15).

**Scheme 16:**
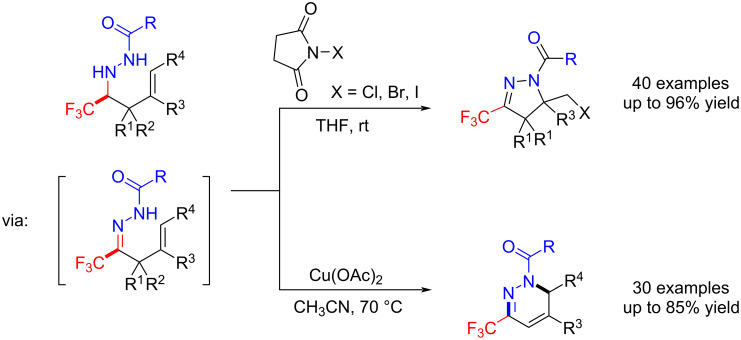
Cascade oxidation/cyclization reactions of trifluoromethylated homoallylic acylhydrazines.

Among these fluorinated products, the trifluoromethylated homoallylic acylhydrazines were easily transformed to CF_3_-substituted pyrazolines and 1,6-dihydropyridazines via a cascade oxidation/cyclization with NXS or Cu(OAc)_2_. Notably, some of the resultant CF_3_-substituted 1,6-dihydropyridazines exhibited aggregation-induced emission [102–103] (Scheme 16).

**Scheme 17:**
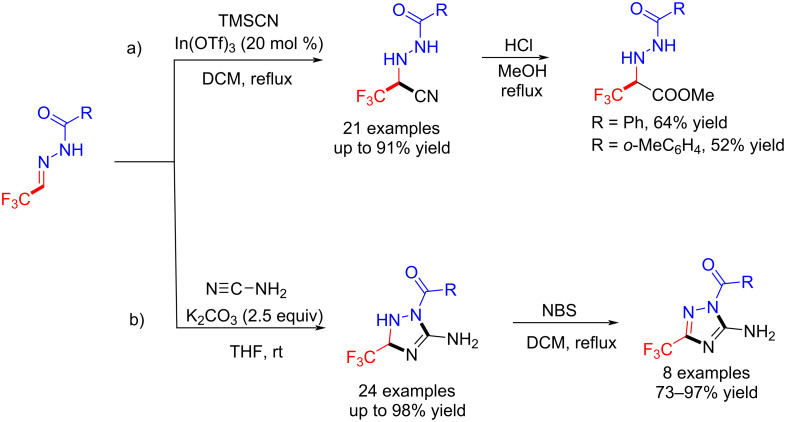
Synthesis of trifluoromethylated cyanohydrazines and 3-trifluoromethyl-1,2,4-triazolines.

The hydrocyanation of acylhydrazones is an important method for the preparation of α-hyrazino acids. Hu et al. reported a Lewis acid-catalyzed hydrocyanation of trifluoromethylated acylhydrazones, in which the product was the precursor for the preparation of chiral fluorinated amino acids [104] (Scheme 17a).

Meanwhile, Hu et al. provided a novel and efficient process for the synthesis of polysubstituted 3-trifluoromethyl-1,2,4-triazolines and their derivatives via tandem 1,2-addition/cyclization reactions between trifluoromethyl acylhydrazones and cyanamide [105] (Scheme 17b).

**Scheme 18:**
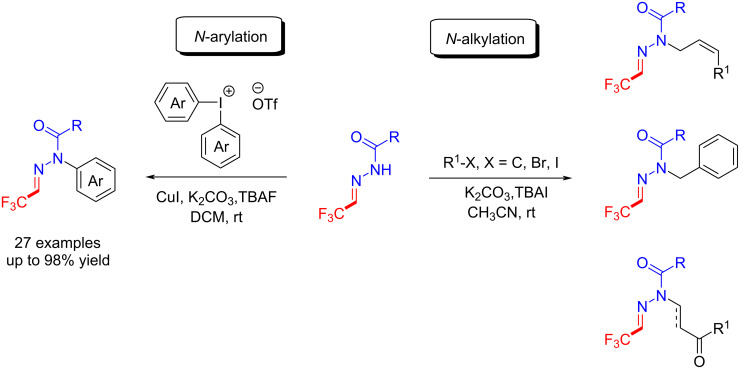
*N*-Arylation and *N*-alkylation of trifluoromethyl acylhydrazones.

Afterwards, Hu et al. developed a method for the *N*-arylation and *N*-alkylation of trifluoromethyl acylhydrazones with diaryliodonium salts and alkyl halides under basic conditions, and expanded the synthetic method to N-substituted acylhydrazones [106–107] (Scheme 18).

**Scheme 19:**
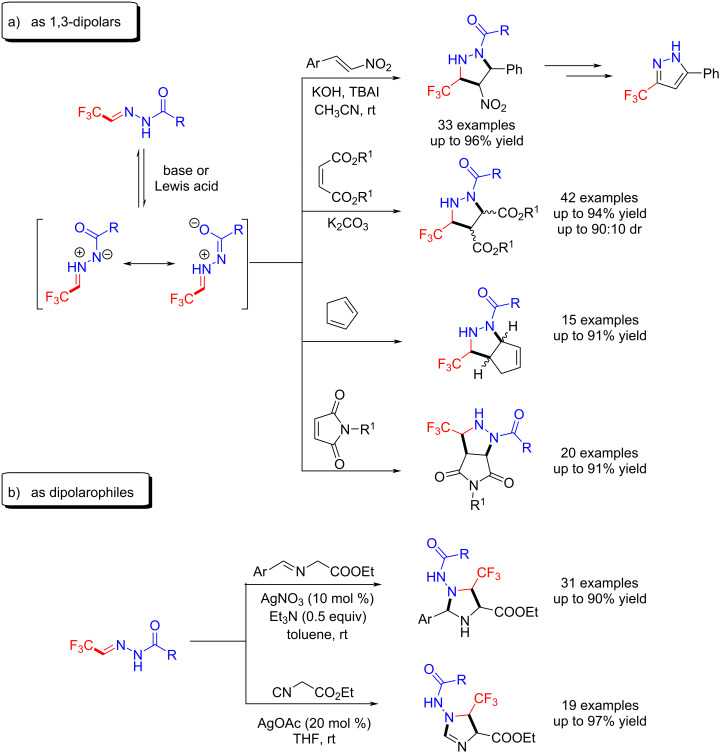
[3 + 2]-Cycladditions of trifluoromethyl acylhydrazones.

In the early development of 1,3-dipolar cycloadditions of azomethine imines, the acyclic azomethine imines were unstable and their in situ preparation required Brønsted acid or thermal activation [108–110]. Besides, pyrazolidine and pyrazoline compounds are highly valuable hereocycles which are found in many natural products and bioactive compounds. Among them, CF_3_-substituted pyrazolidines have already been shown to be highly bioactive [111–113]. Thus, Hu et al. chose trifluoromethyl acylhydrazones as 1,3-dipolar agents to react with β-nitrostyrenes [114], maleates [115], cyclopentadiene [116] and maleimides [117] for the synthesis of CF_3_-substituted pyrazolidine derivatives. These reactions were conducted under basic conditions and in the presence of Cu(OTf)_2_ (Scheme 19a).

As an extension of their trifluoromethyl acylhydrazone synthesis, Hu et al. reported that trifluoromethyl acylhydrazones react with azomethine ylides [118] and ethyl isocyanoacetate [119] to generate trifluoromethylated imidazolidines. They demonstrated then that trifluoromethyl acylhydrazones act as dipolarophiles in the [3 + 2]-cycladditions (Scheme 19b).

## Conclusion

Fluorine-containing molecules have attracted widespread attention as important components of agrochemicals, pharmaceuticals, and advanced materials. Abundant and fruitful progress in the applications of fluoromethylated hydrazones and acylhydrazones in recent years have been summarized and discussed. The resultant fluorinated building blocks provided a facile and rapid approach to directly construct valuable nitrogen-containing fluorinated compounds. Apart from the regular involvement of addition and annulation reactions, the exploitation of more in-depth applications of fluoromethylated hydrazones and acylhydrazones to synthesize natural product analogues and fluorinated drugs is highly desirable. These methods should encourage the introduction of these difluoromethylated nitrogen-containing building blocks in future bioactives discovery programs.
